# Characterization of Aspergillus niger Isolated from the International Space Station

**DOI:** 10.1128/mSystems.00112-18

**Published:** 2018-09-18

**Authors:** Jillian Romsdahl, Adriana Blachowicz, Abby J. Chiang, Nitin Singh, Jason E. Stajich, Markus Kalkum, Kasthuri Venkateswaran, Clay C. C. Wang

**Affiliations:** a Department of Pharmacology and Pharmaceutical Sciences, School of Pharmacy, University of Southern California, Los Angeles, California, USA; b Biotechnology and Planetary Protection Group, Jet Propulsion Laboratory, California Institute of Technology, Pasadena, California, USA; c Department of Molecular Immunology, Beckman Research Institute of City of Hope, Duarte, California, USA; d Department of Microbiology and Plant Pathology and Institute of Integrative Genome Biology, University of California—Riverside, Riverside, California, USA; e Department of Chemistry, Dornsife College of Letters, Arts, and Sciences, University of Southern California, Los Angeles, California, USA; University of California, Berkeley

**Keywords:** *Aspergillus niger*, International Space Station, phylogenetic analysis, proteomics

## Abstract

The initial characterization of the Aspergillus niger isolate JSC-093350089, collected from U.S. segment surfaces of the International Space Station (ISS), is reported, along with a comparison to the extensively studied strain ATCC 1015. Whole-genome sequencing of the ISS isolate enabled its phylogenetic placement within the A. niger/*welwitschiae*/*lacticoffeatus* clade and revealed that the genome of JSC-093350089 is within the observed genetic variance of other sequenced A. niger strains. The ISS isolate exhibited an increased rate of growth and pigment distribution compared to a terrestrial strain. Analysis of the isolate’s proteome revealed significant differences in the molecular phenotype of JSC-093350089, including increased abundance of proteins involved in the A. niger starvation response, oxidative stress resistance, cell wall modulation, and nutrient acquisition. Together, these data reveal the existence of a distinct strain of A. niger on board the ISS and provide insight into the characteristics of melanized fungal species inhabiting spacecraft environments.

**IMPORTANCE** A thorough understanding of how fungi respond and adapt to the various stimuli encountered during spaceflight presents many economic benefits and is imperative for the health of crew. As A. niger is a predominant ISS isolate frequently detected in built environments, studies of A. niger strains inhabiting closed systems may reveal information fundamental to the success of long-duration space missions. This investigation provides valuable insights into the adaptive mechanisms of fungi in extreme environments as well as countermeasures to eradicate unfavorable microbes. Further, it enhances understanding of host-microbe interactions in closed systems, which can help NASA’s Human Research Program maintain a habitat healthy for crew during long-term manned space missions.

## Abstract (precis)

A thorough understanding of how fungi respond and adapt to the various stimuli encountered during spaceflight presents many economic benefits and is imperative for the health of crew. As A. niger is a predominant ISS isolate frequently detected in built environments, studies of A. niger strains inhabiting closed systems may reveal information fundamental to the success of long-duration space missions. This investigation provides valuable insights into the adaptive mechanisms of fungi in extreme environments as well as countermeasures to eradicate unfavorable microbes. Further, it enhances understanding of host-microbe interactions in closed systems, which can help NASA’s Human Research Program maintain a habitat healthy for crew during long-term manned space missions.

## INTRODUCTION

Throughout the history of human space exploration, filamentous fungi have traveled with us and are omnipresent on spacecraft (1–3). Microorganisms have been reported to cause biodegradation of structural spacecraft components, resulting in decreased integrity of spacecraft hardware (2). Microbial infections also constitute a major health risk for astronauts, especially in closed environments where the combined stresses of sleep disruption, microgravity, and high levels of radiation may further compromise the human immune system (2, 4). Studies have suggested that microbial virulence and antimicrobial resistance increase in response to spacecraft environments (5–7). Other reports have associated the abundance of filamentous fungus in indoor environments with allergies and invasive infections (8, 9). Additionally, fungi produce a myriad of bioactive secondary metabolites (SMs) in response to environmental stressors, and while many SMs have diverse therapeutic and industrial applications, others are toxins and can have detrimental effects on human health (10). As we set our exploration sights beyond low-Earth orbit, a thorough understanding of how fungi respond and adapt to the various stimuli encountered during spaceflight is critical to the success of long-term space travel.

Microorganisms inhabiting the International Space Station (ISS) are exposed to microgravity and have increased exposure to high-energy radiation as a result of being outside Earth’s protective atmosphere (11). In general, it is thought that microgravity alters biological processes by initially altering the physical forces acting on the cell and its environment. This results in decreased transfer of extracellular nutrients and metabolic by-products, causing the cell to be exposed to a completely different chemical environment (11). The inside cabin of the ISS is exposed to a complex radiation environment (12), at levels that are not fungicidal (13), permitting fungi to thrive. Radiation primarily interacts with biological systems through the ionization and excitation of electrons in molecules, and its strong mutagenic properties result in an increased rate of biological evolution (14). Further, radiation can have many harmful effects on biological systems, which results in the development of adaptive responses. Fungi inhabiting spacecraft are also forced to acclimate to reduced nutrient availability, as the National Aeronautics and Space Administration (NASA) routinely performs stringent microbial monitoring and remediation on the ISS (15).

Aspergillus niger was reported to be the predominant species isolated in one ISS microbial monitoring study (15), which is consistent with its frequent detection in built environments (16). A. niger is a melanized fungal species that is ubiquitous in nature and commonly used in biotechnology industries as a production host for citric acid and enzymes (17). Despite the recurring detection of A. niger in spacecraft environments, investigations into its genetic alteration and gene expression modulation under ISS conditions have not been carried out. Although A. niger is less pathogenic to humans than other *Aspergillus* species, such as A. fumigatus and A. flavus (17), it has been associated with ear infections and can cause invasive pulmonary aspergillosis in immunocompromised patients (18). This enhances the need for studies to understand how A. niger responds and adapts to the environment of the ISS, where microgravity might play a role in compromising the human immune system (2, 4). Additionally, melanized fungi are highly resistant to ionizing radiation and respond to radiation with enhanced growth and upregulation of many proteins (19, 20), some of which may provide important insight into the adaptive evolutionary mechanism of melanized fungal species.

The objective of this study was to investigate a strain of A. niger isolated from surfaces of the ISS, with the aim to characterize its molecular phenotype. Although it has been well established that fungi are ubiquitous on spacecraft (1–3, 15), very few studies have been conducted to characterize fungi isolated from the ISS (21). Given that melanin production in fungi is considered an evolution-derived trait to confer radiation resistance (19, 22), the present study of a melanized fungus that has inhabited the ISS may reveal important insights into the key traits necessary to withstand such environments. Our work investigated differences of the ISS A. niger isolate from Earth isolates to better understand the characteristics of strains isolated from the space station built environment. Due to the significance of secondary metabolic processes in filamentous fungi (23), A. niger ATCC 1015 was used as a terrestrial reference strain for physiologic and proteomic analyses because its SM profile has been thoroughly characterized (24), and we aim to build on this work by investigating SM production in JSC-093350089.

## RESULTS

### Identification of A. niger sampled from the ISS.

**FIG 1:**
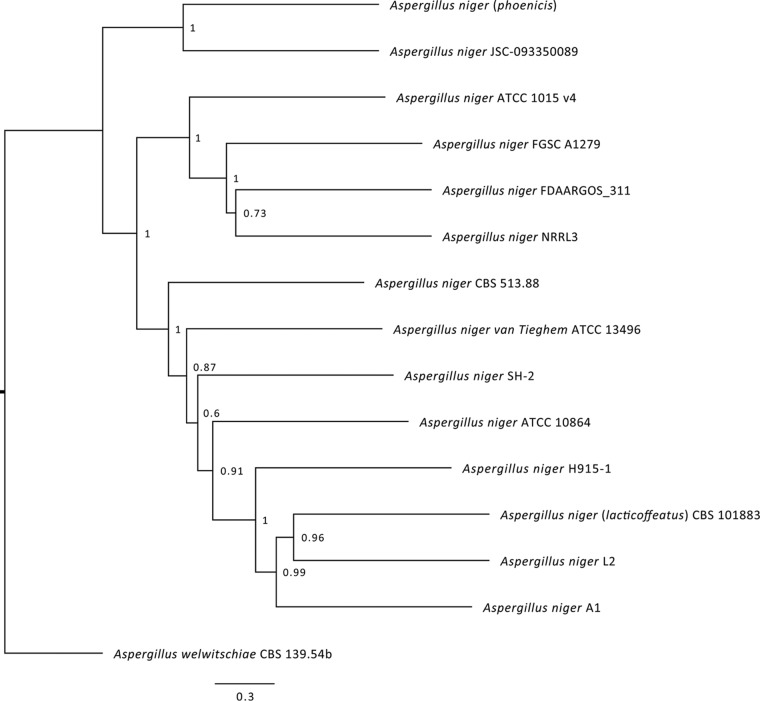
Phylogenetic characterization of JSC-093350089 displaying its relative placement within the A. niger/*welwitschiae*/*lacticoffeatus* clade.

Sampling of surfaces on the ISS during microbial monitoring surveys resulted in the isolation of numerous bacterial and fungal strains (15). A strain of A. niger, JSC-093350089, identified by morphological characteristics and verified by internal transcribed spacer (ITS) region sequencing, was used for this study. This strain was isolated by swabbing surface materials on the U.S. segment of the ISS. Due to the nature of this sampling method, it is impossible to know the exact duration of time that this strain was on the ISS. The 36.08-Mb genome sequence of JSC-093350089 was generated using whole-genome paired-end sequencing (WGS), which was further improved to high-quality assemblies of 223 scaffolds possessing 12,532 coding sequences and 287 tRNAs. The JSC-093350089 genome was similar in size to other A. niger genomes, which typically range from 34.0 to 36.5 Mb (25–27). To further verify the identity of JSC-093350089 and place it into the larger context of the A. niger/*welwitschiae*/*lacticoffeatus* clade, phylogeny was assessed using maximum likelihood (Fig. 1). Of the A. niger strains surveyed, the ISS isolate displayed the closest phylogenetic relationship to A. niger (*phoenicis*). Compared to ATCC 1015, an industrial strain used for citric acid production (25), and CBS 513.88, an ancestor of the A. niger strains used industrially for enzyme production (27), it differed by 37,548 and 39,433 variants, respectively.

### Visual characterization and growth rates of JSC-093350089 *in vitro*.

**FIG 2:**
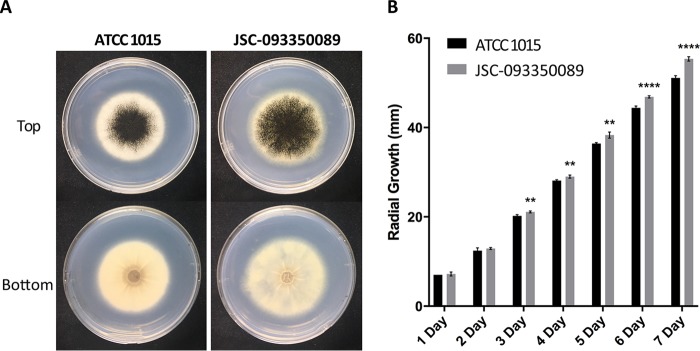
*In vitro* growth of JSC-093350089 compared to ATCC 1015. (A) Growth on GMM at 30°C after 7 days, showing colony morphology and color. (B) Radial growth at 30°C on GMM. Statistical analyses were performed by multiple *t* tests, corrected for multiple comparisons using the Holm-Sidak method. **, *P* value = 0.0021 to 0.0002; **** *P* value < 0.0001.

The basic physiological phenotype of JSC-093350089 was investigated on glucose minimal medium (GMM) agar plates. Visual characterization of centrally inoculated GMM plates revealed differences in pigment distribution and colony diameter after 7 days of growth (Fig. 2A). JSC-093350089 colony size appeared larger, and pigment had spread to the periphery of the colony in a shorter time than for ATCC 1015. Assessment of radial growth rates revealed that the ISS strain grew at a significantly higher rate than ATCC 1015 after 3 days of growth (Fig. 2B).

### Overview of proteome analysis.

**FIG 3:**
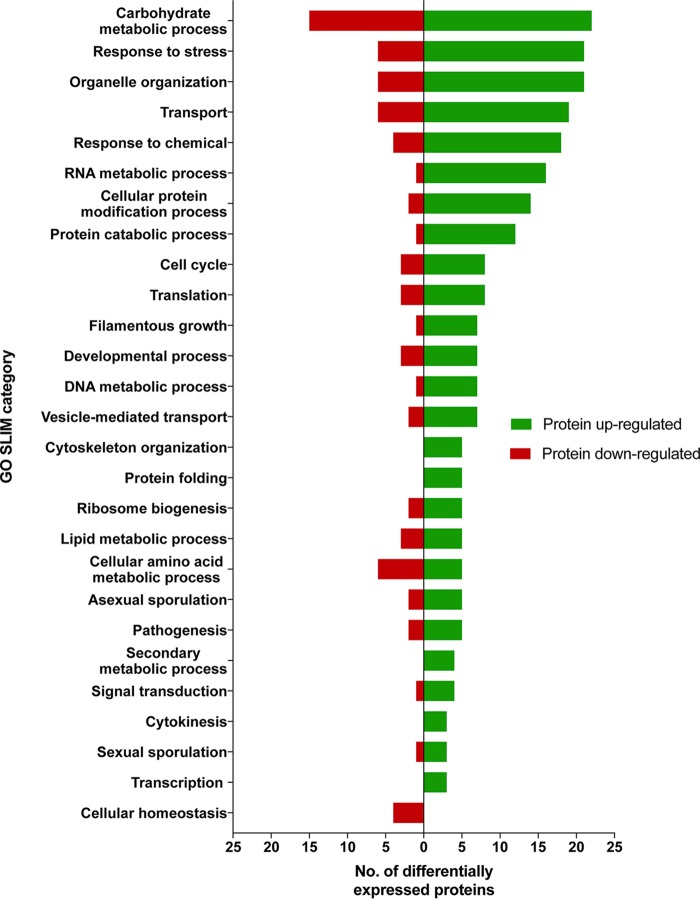
Biological process GO Slim categories of differentially expressed proteins. Differentially enriched proteins (FC >|2|, *P* < 0.05) were mapped to terms representing various biological processes using AspGD Gene Ontology (GO) Slim Mapper.

To investigate the differences in the proteomes of JSC-093350089 and ATCC 1015, total protein was extracted from each strain and subjected to tandem mass tag (TMT) labeling, followed by liquid chromatography-mass spectrometry (LC-MS) analysis. All MS data were analyzed using the Proteome Discoverer with the Sequest-HT search engine against the A. niger CBS 513.88 protein database (NCBI). The CBS 513.88 protein database was used because it has been extensively annotated and enabled subsequent functional analysis using the AspGD Gene Ontology (GO) Slim Mapper tool. The abundance ratios for all proteins were normalized to ATCC 1015, which resulted in the identification of 218 proteins with increased abundance and 109 proteins with decreased abundance (fold change [FC] >|2|, *P* < 0.05), in JSC-093350089, relative to ATCC 1015 (see Table S1 in the supplemental material). Distribution of AspGD GO Slim terms among differentially expressed proteins is displayed in Fig. 3. Many proteins that exhibited increased abundance in JSC-093350089 were involved with carbohydrate metabolic processes (10.1% of all upregulated proteins), response to stress (9.6%), organelle organization (9.6%), and transport (8.7%). Proteins involved in cytoskeleton organization, protein folding, secondary metabolic processes, and transcription were associated with only increased protein abundance in JSC-093350089, while proteins involved in cellular homeostasis were associated with only decreased protein abundance in JSC-093350089. GO Slim term enrichment analysis was conducted using FungiDB (28), which identified significantly overrepresented proteins that exhibited increased abundance in the proteome of JSC-093350089 (Table S2). Significantly overrepresented GO Slim terms included carbohydrate metabolic processes (4.7% of background genes with this term), cellular component assembly (5.3%), catabolic processes (4.1%), protein complex assembly (6.4%), and response to stress (3.2%).

10.1128/mSystems.00112-18.1 TABLE S1 Relative abundance ratios for proteins identified in JSC-093350089 compared to ATCC 1015. Download Table S1, XLSX file, 0.1 MB. Copyright © 2018 Romsdahl et al. 2018 Romsdahl et al. This content is distributed under the terms of the Creative Commons Attribution 4.0 International license.

10.1128/mSystems.00112-18.2 TABLE S2 GO Slim enrichment analysis of significantly overrepresented biological processes with increased protein abundance in proteome of JSC-093350089 compared to ATCC 1015. Analysis conducted using FungiDB GO Slim enrichment analysis tool (28). Download Table S2, XLSX file, 0.04 MB. Copyright © 2018 Romsdahl et al. 2018 Romsdahl et al. This content is distributed under the terms of the Creative Commons Attribution 4.0 International license.

### Differential abundance of cell wall modulation proteins.

The proteome of JSC-093350089 revealed differential levels of cell wall modulation proteins (Table 1). Conidia of A. niger possess a relatively thick cell wall made of a network of carbohydrates, including β-glucans, chitin, α-glucans, galactomannan, and galactosaminogalactan, with an outer cell wall layer consisting of complex melanin pigments (29). The polyketide synthase AlbA (An09g05730), which is required for the production of 1,8-dihydroxynaphthalene-melanin (DHN-melanin) in A. niger (24), was over 2-fold more enriched in JSC-093350089 than in ATCC 1015. The protein abundance of hydrophobin Hyp1 (An07g03340) was nearly 3-fold higher than in ATCC 1015. RodA, the homologue of Hyp1 in A. nidulans, has been reported to play a role in biofilm formation and efficient deconstruction of cell wall polysaccharides (30). Its homologue in A. fumigatus was shown to enhance fungal virulence by masking dectin-1- and dectin-2-mediated recognition of conidia *in vivo* (31).

**TABLE 1:** Relative abundance of cell wall modulation proteins

ORF^*b*^	Protein	CAZy family	Description	LogFC^*a*^
An16g02910		GH92	α-Mannosidase	3.53
An02g09050	GelG	GH72	β-1,3-Glucanotransferase	2.99
An14g04240		GH92	α-1,2-Mannosidase	2.25
An07g08640	AgnB	GH71	α-1,3-Glucanase	2.23
An13g01260		GH92	α-1,2-Mannosidase	2.15
An11g03340	AamA	GH13	Acid α-amylase	2.01
An11g06080		GH3	β-Glucosidase	1.68
An06g01530	Scw4	GH17	β-Glucanase	1.6
An01g11660	CbhB	GH7	1,4-β-Glucan cellobiohydrolase	1.53
An02g13180	BgxB	GH55	β-1,3-Glucanase	1.48
An07g03340	Hyp1		Spore wall fungal hydrophobin	1.47
An01g09290	TraB	GH37	Trehalase	1.27
An09g05730	AlbA		Polyketide synthase	1.24
An08g11070	SucA	GH32	Invertase	1.23
An08g08370		GH92	α-Mannosidase	1.19
An14g04190	GbeA	GH13	1,4-α-Glucan branching enzyme	1.18
An01g09960	XlnD	GH3	β-d-Xylosidase	1.06
An14g05340	UrghB	GH105	Rhamnogalacturonyl hydrolase	−1
An10g00400	GelA	GH72	β-1,3-Glucanotransferase	−1.04
An16g06800	EglB	GH5	Endoglucanase	−1.13
An09g03100	AgtA	GH13	GPI-anchored α-glucanosyltransferase^*c*^	−1.2
An04g06930	AmyC	GH13	α-Amylase	−1.22
An18g03570	BglA	GH3	β-Glucosidase	−1.22
An01g12150	LacA	GH35	β-Galactosidase	−1.4
An02g00610		GH2	β-Glucuronidase	−1.41
An12g08280	InuE	GH32	Exoinulinase	−1.5
An11g02100		GH1	β-Glucosidase	−1.54
An14g01770		GH3	β-Glucosidase	−1.54
An11g00200		GH3	β-Glucosidase	−1.69
An07g08950	EglC	GH5	Endoglucanase	−1.82
An15g03550		GH43	Endoarabinase	−1.91

a Log_2_ fold change of JSC-093350089 compared to ATCC 1015 (*P* < 0.05).

b ORF, open reading frame.

c GPI, glycosylphosphatidylinositol.

Differential expression was observed for a number of genes encoding glycoside hydrolases, which were identified using the CAZy database (http://www.cazy.org/) (32). The starvation-induced cellobiohydrolase CbhB (An01g11660), which is regulated by XlnR (33), exhibited levels nearly 3-fold higher in the proteome of JSC-093350089 than in ATCC 1015 (34). XlnR is a transcriptional activator that regulates xylanolytic, endoglucanase, and cellobiohydrolase gene expression in A. niger (33, 35). Increased protein abundance was observed for β-d-xylosidase XlnD (An01g09960), which is also regulated by XlnR (35). Decreased protein abundance was observed for XlnR-regulated β-galactosidase LacA (An01g12150), which is exclusively expressed on xyloglucan-derived substrates (36). Similarly, XlnR-regulated endoglucanase EglC (An07g08950), which exhibits its greatest activity toward xyloglucan, also displayed decreased protein abundance (37). Other starvation-induced cell wall degradation glycoside hydrolases enriched in JSC-093350089 included α-1,3-glucanase AgnB (An07g08640) and β-glucanase Scw4 (An06g01530). Differential abundance of glycoside hydrolases involved in starch utilization was also observed. Extracellular acid α-amylase AamA (An11g03340), which plays a role in starch degradation and is regulated by starch degradation regulator AmyR (38), was present in JSC-093350089 at levels 4-fold higher than that in ATCC 1015. Four of the five enzymes in the family of GH92, which consists of mannosidases, displayed increased abundance in the proteome of JSC-093350089. These GH92 proteins included An08g08370, An13g01260, An14g04240, and An16g02910.

### Differential abundance of stress response proteins.

Our study also revealed differential abundance of proteins involved in the stress response of A. niger (Table 2). Heat shock proteins, including DnaK-type molecular chaperone Ssb2 (An16g09260) and An06g01610, were present in JSC-093350089 at levels 2-fold and 5-fold higher than that of ATCC 1015, respectively. An06g01610 is very similar to late embryogenesis abundant (LEA)-like Hsp12 of Saccharomyces cerevisiae and has been reported to stabilize the plasma membrane (39). Increased protein abundance was observed for the serine/threonine protein kinase Srk1 (An07g07970) and the mitogen-activated protein kinase SakA (An08g05850), which have been reported to mediate cell cycle arrest and mitochondrial function in response to oxidative stress (40). Other proteins that exhibited higher levels in JSC-093350089 included the oxidative stress protein Svf1 (An18g02900) and An02g07350, which encodes a protein homologous to group 3 LEA proteins responsible for mitigating stress-induced damage, such as protecting seeds from drought (41, 42). The catalase An12g10720 was present at levels 13-fold higher than that of ATCC 1015. Increased abundance was also observed for the stress response nuclear envelope protein Ish1, whose expression has been reported to increase in response to glucose starvation and osmotic stress (43).

**TABLE 2:** Relative abundance of stress response proteins

ORF	Protein	Description	LogFC^*a*^
An12g10720		Catalase	3.71
An06g01610		Heat shock protein	2.51
An02g07350		LEA domain protein	1.95
An16g04420	Ish1	Stress response protein	1.53
An08g05850	SakA	MAP kinase^*b*^	1.5
An18g02900	Svf1	Survival factor 1	1.43
An07g07970	Srk1	Serine/threonine protein kinase	1.21
An16g09260	Ssb2	Heat shock protein	1.09

a Log_2_ fold change of JSC-093350089 compared to ATCC 1015 (*P* < 0.05).

b MAP, mitogen-activated protein.

## DISCUSSION

In the current study, the molecular phenotype of a strain of A. niger isolated from the ISS was characterized. Despite its frequent detection in built environments, this is the first investigation into the “omic” differences of an ISS A. niger isolate from an Earth strain. As the frequency and duration of manned space missions increase, investigations into how fungi respond and adapt to various stimuli encountered during spaceflight are imperative for the health of crew and present many economic benefits. Further, such studies provide insight into the adaptive evolutionary mechanism of melanized fungal species and the biological alterations of microbes isolated from extreme spaceflight environments.

The genome of JSC-093350089 was within the genetic variation of other A. niger strains, suggesting that its ability to survive and proliferate in a spacecraft environment is not contingent on enhanced genetic variance. This finding is consistent with a previous report on the genetic variance of ISS *Aspergillus* isolates (21). To further understand the effect of microgravity and enhanced irradiation on fungal genomics, future studies should investigate the same strain grown under both space and ground conditions to quantify and identify any mutations that may result from life on the ISS. Additional sequencing of terrestrial A. niger strains will also be important to better identify the donor population of the strain and further isolate the sequence variation that is specific to ISS-derived strains.

One characteristic of the ISS isolate was increased protein abundance of AlbA, a key biosynthesis enzyme involved in the production of DHN-melanin in A. niger. While A. niger historically has black conidia due to its high melanin content, the A. niger Δ*albA* mutant was reported to display a white or colorless conidial phenotype (24). This is consistent with reports that fungi isolated from high-radiation environments exhibit increased melanin production. One study found that A. niger strains occupying the south-facing slope of the “Evolution Canyon” in Israel, which receives 200% to 800% higher solar radiation than the north-facing slope, produced three times more melanin than did strains isolated from the north-facing slope (44). It is reasonable to presume that increased melanin production is a key adaptive response to the enhanced irradiation environment of the ISS, as there is considerable evidence that melanized fungi are highly resistant to ionizing radiation under experimental conditions (19, 45). In fact, it has been reported that exposure of melanin to ionizing radiation alters its electronic properties, and melanized fungal cells exhibit increased growth rates following exposure to ionizing radiation (46).

Interestingly, the ISS isolate exhibited a higher growth rate than the terrestrial strain, which is consistent with previous reports of enhanced growth in melanized fungi following radiation exposure and *Aspergillus* and *Penicillium* species recovered from the ISS and Mir space stations (19, 21, 46, 47). Although this finding cannot be definitively attributed to the isolation environment, it is conceivable that rapid growth may confer a selective advantage in environments operating under strict microbial monitoring procedures. The reported increase in colony pigmentation distribution may point to enhanced melanin production in the ISS isolate, as the AlbA protein was 2-fold more enriched than in the terrestrial strain. However, the ability to rapidly spread pigment to the periphery of the colony may offer additional modes of protection from high levels of radiation present in spacecraft.

The ISS isolate displayed general hallmarks of carbon starvation. During starvation, A. niger produces a myriad of glycoside hydrolases that facilitate the release of nutrients from biopolymers and the recycling of cell wall components to generate energy and building blocks that can be used for maintenance and conidiogenesis (48, 49). The observed enrichment of starvation-induced nutrient acquisition enzymes may be the result of adaptation to the low-nutrient environment that exists as a result of stringent microbial monitoring and remediation by NASA (15). The same may also be true for the increased abundance of the glycoside hydrolase AamA. AamA is highly upregulated in growing hyphae at the periphery of mycelium (50), and following secretion from exploring hyphae, AamA degrades starch into small molecules that can be taken up by the fungus to serve as nutrients. The significant enrichment of AamA suggests that JSC-093350089 can utilize starch encountered during colonization more efficiently than ATCC 1015, which may have conferred a selective advantage in the low-nutrient spacecraft environment.

During spaceflight, ionizing radiation can generate reactive oxygen species (ROS) via the hydrolysis of intracellular water, which can result in oxidative damage to DNA, proteins, lipids, and other cell components (51). Accordingly, catalase was among the highest-upregulated proteins in the ISS isolate, which degrade H_2_O_2_ and therefore play a major role in curtailing oxidative stress (52, 53). This is consistent with previous reports that spaceflight induces the expression of oxidative stress resistance genes in microbes, animals, and astronauts (20, 54–59), and increased susceptibility to ionizing radiation has been observed in S. cerevisiae strains lacking cytosolic catalase (60, 61). Similarly, increased abundance was also observed for kinases that mediate key biological processes in response to oxidative stress (40). The high levels of oxidative stress response proteins, as found in this study, are consistent with the observed response of the melanized yeast Wangiella dermatitidis following exposure to ionizing radiation (20). On the other hand, increased resistance to oxidative stress may be a response to microgravity, as low-shear modeled microgravity has induced such a response in bacteria (62).

This study has revealed the existence of a distinct strain of A. niger on board the ISS that exhibited differential growth and conidiation patterns compared to a terrestrial strain. Proteomic analysis revealed significant differences in the phenotype of JSC-093350089 that included enrichment of proteins involved in the A. niger starvation response, oxidative stress resistance, cell wall modulation, and nutrient acquisition. Given the ubiquity of A. niger in nature along with its genetic diversity among sequenced strains (25, 26), it is not surprising that JSC-093350089 exhibited a distinct molecular phenotype, and more studies will reveal if the observed phenotype is widespread for other A. niger strains isolated from the ISS. Since most of the microgravity-induced response studies were carried out utilizing opportunistic pathogens of bacteria and yeast, the “omics” characterization of ISS A. niger, a saprophyte, which exhibited higher melanin content than its Earth counterparts, could be a model to elucidate molecular mechanisms involved in microbial adaptation to the ISS environment. Developing countermeasures to eradicate problematic microorganisms that adapt to unfavorable conditions would help NASA’s Human Research Program in planning for long-duration manned missions. Additionally, such analyses will further our understanding of the molecular pathways that define host-microbe interactions, thus enabling development of suitable cleaning strategies to maintain the health of habitat and coliving crew for future missions.

## MATERIALS AND METHODS

### Isolation and identification of the ISS A. niger isolate.

Surface samples were collected from the U.S. segment of the ISS using the Surface Sampling kit (SSK) (NASA, 2011). Microbes were removed from surfaces using a swab and sterile saline solution (0.85% sodium chloride) and were transported to Earth for analyses. Materials retrieved from the swabs were subsequently inoculated onto potato dextrose agar (PDA) supplemented with chloramphenicol. The PDA plates were incubated at ambient cabin temperature (28° to 37°C) for 5 days. The fungal colonies that exhibited growth were further purified and stored at −80°C in sterile glycerol stock until further analyses. When required, fungal isolates were revived on PDA medium, and DNA from pure cultures was extracted (UltraPure DNA kit [Mo Bio, Carlsbad, CA]). An approximately 600-bp region consisting of ITS 1, 5.8S, and ITS 2 of the isolated fungal DNA was PCR amplified, using primers ITS1F (5′ TTG GTC ATT TAG AGG AAG TAA 3′) and Tw13 (5′ GGT CCG TGT TTC AAG ACG 3′) (63) and following the established protocol (64). The UNITE database was used to determine the closest similarity to ITS sequences of fungal type strains (65). The identity of the ISS isolate was subsequently confirmed by WGS.

### Genome sequencing, assembly, and annotation.

Extracted DNA was sent to the Macrogen clinical laboratory (Macrogen Inc., Rockville, MD, USA) for WGS. Library preparation was carried out using the Illumina Nextera kit (random fragmentation, adapter ligation, and cluster generation) and quantified with Quant-iT double-stranded DNA (dsDNA) high-sensitivity assays. Generated libraries were sequenced with 100-bp paired-end sequencing protocols on the Illumina HiSeq 2500 platform. Raw data images were produced utilizing HCS (HiSeq Control Software v2.2.38) for system control, and base calling (BCL) was done through an integrated primary analysis using Real Time Analysis software v1.18.61.0. The BCL binary was converted into FASTQ utilizing Illumina package bcl2fastq (v1.8.4). The NGS QC Toolkit version 2.3 (66) was used to filter the data for high-quality vector- and adapter-free reads for genome assembly (cutoff read length for high quality, 80%; cutoff quality score, 20), and 22,769,466 vector filter reads were obtained after the quality check. High-quality vector-filtered reads were used for *de novo* assembly with the MaSuRCA genome assembler (k-mer size, 70) (67). The final assembly consisted of 223 scaffolds with a total size of 36,079,011 bp (∼100×). The *N*_50_ scaffold length was 543,773 kb, and the largest scaffold was 1,390.254 kb. There was no random “N” joining of the contigs to maintain high assembly quality. Quality check of the final assembly was performed using the quality assessment tool for genome assemblies (QUAST) (68). The number of N’s detected was less than 12 per 100 kb, which represents very good assembly.

Genome annotation was performed with funannotate (69) (https://github.com/nextgenusfs/funannotate; v1.3.0-beta), which utilizes a combination of *ab initio* gene prediction tools (70, 71) and experimental evidence including proteins and transcriptome sequencing (RNA sequencing) and a consensus gene calling with EvidenceModeler (72).

### Phylogenetic analysis.

Phylogenetic analysis of A. niger strains was performed by identifying conserved protein coding genes in available A. niger genomes and close relatives. These data were obtained by downloading public sequence data from NCBI and the Department of Energy’s Joint Genome Institute (JGI) Mycocosm. The assemblies for strains A1, ATCC 10864, An76, FDAARGOS 311, FGSC A1279, H915-1, L2, and SH-2 were downloaded from the NCBI Assembly Archive. The strains FDAARGOS 311 and An76 already had deposited annotations and were downloaded directly. For the remaining strains, gene prediction with Augustus (v 3.2.2) (70) used the pretrained model ‘aspergillus_niger_jsc_093350089’ generated from the genome annotation procedure. This parameter set is deposited in https://github.com/hyphaltip/fungi-gene-prediction-params. Additional strains with annotation from JGI were downloaded (ATCC 1015, DSM 1, CBS 513.88, and NRRL 3) along with related species (A. niger van Tieghem ATCC 13496, A. welwitschiae CBS 139.54b, A. phoenicis, A. lacticoffeatus CBS 101883, and A. brasiliensis CBS 101740). Coding sequences were obtained, translated into proteins, and searched for a conserved set of 71 protein coding gene markers, “AFTOL_70,” as part of the 1000 Fungal Genomes project (https://github.com/1KFG/Phylogenomics_HMMs). These markers were searched using PHYling (https://github.com/stajichlab/PHYling_unified), which first searches for conserved markers using HMMsearch followed by extraction of best hits and concatenated alignment of all the orthologous matches. A back-translated alignment of coding sequences was produced from the input proteins in order to resolve the closely related strains in this data set. A phylogenetic tree was inferred from the coding sequence tree using IQTREE (v1.6.3) first by identifying a partition scheme with -m TESTMERGE -st CODON parameters followed by a tree inference using the options -st CODON -bb 1000 -spp Partition.txt to infer the tree and obtain branch support with ultrafast bootstrapping on the reduced partition parameters under a codon model in IQTREE (73–75). To identify the number of single nucleotide variations occurring between JSC-093350089 and both ATCC 1015 and CBS 513.88, variants were called using the Harvest suite’s Parsnp tool (76).

### Growth conditions.

JSC-093350089 and ATCC 1015 were cultivated on 10-cm petri dishes containing 25-ml glucose minimal medium (GMM) agar plates (6 g/liter NaNO_3_, 0.52 g/liter KCl, 0.52 g/liter MgSO_4_·7H_2_O, 1.52 g/liter KH_2_PO_4_, 10 g/liter d-glucose, 15 g/liter agar supplemented with 1 ml/liter of Hutner’s trace elements) with a cellophane membrane on top, on which the fungus was grown. Unless otherwise specified, 1 × 10^7^ conidia per petri dish (*D* = 10 cm) were inoculated into each medium and incubated at 30°C for 5 days.

### Physiological analysis.

Growth rates were assessed by centrally inoculating 1 × 10^4^ conidia on GMM plates in replicates of 5 and measuring radial growth at the same time each day. Statistical analyses were performed using multiple *t* tests and corrected for multiple comparisons using the Holm-Sidak method. Photos depicting morphological differences were taken after 7 days.

### Protein extraction.

Mycelia from GMM agar plates were collected and stored at −80°C prior to protein extraction. For protein extraction, the lysis buffer consisted of 100 mM triethylammonium bicarbonate (TEAB) with 1× Halt protease inhibitor cocktail (100×), with the final concentration of each component being 1 mM AEBSF [4-(2-aminoethyl)benzenesulfonyl fluoride hydrochloride], 800 nM aprotinin, 50 μM bestatin, 15 μM E64, 20 μM leupeptin, and 10 μM pepstatin A (Thermo Scientific, Rockford, IL) and 200 µg/ml phenylmethylsulfonyl fluoride (Sigma-Aldrich, St. Louis, MO). Mycelia were homogenized directly using Precellys 24 homogenizer (Bertin, Rockville, MD) in which each sample was processed inside a 2-ml cryotube with 0.5-mm glass beads three times (at 4°C and 6,500 rpm for 1 min., repeated 3 times with 15-s pauses in between). The lysed fungi were centrifuged at 17,000 × *g* for 15 min. Protein concentrations in the supernatants were measured by the Bradford assay with albumin for the standard curve (Bio-Rad Laboratories, Inc., Hercules, CA).

### Tandem mass tag (TMT) labeling.

Two hundred micrograms of proteins from each sample was precipitated in 20% trichloroacetic acid (TCA) at 4°C. Protein pellets were obtained by centrifugation (17,000 × *g*), washed with ice-cold acetone, and resuspended in 25 µl TEAB (50 mM final concentration) and 25 µl 2,2,2-trifluoroethanol (TFE) (50% final concentration). Proteins were reduced by adding 1 µl of tris(2-carboxyethyl)phosphine (TCEP; 500 mM) followed by incubation for 1 h at 37°C (10 mM final TCEP concentration). Proteins were alkylated in the presence of iodoacetamide (IAA; 30 mM) in the dark for 1 h at room temperature. A 2.5-µg-per-sample quantity of mass-spectrometry-grade trypsin-LysC (Promega, Madison, WI) was used to digest the peptides overnight at 37°C.

The digested peptides were quantified using the Pierce quantitative colorimetric peptide assay (Thermo Scientific). Forty micrograms of peptides from each specific sample was labeled with the Thermo Scientific TMTsixplex isobaric mass tagging kit (ATCC 1015-GMM with TMT^6^-128, and JSC-GMM with TMT^6^-129) according to the manufacturer’s protocol. The TMT^6^-130 and -131 labels were used as a reference that contained an equal amount of the peptides from each of the four samples. All labeled-peptide mixtures were combined into a single tube, mixed, and fractionated using the Thermo Scientific Pierce high-pH reversed-phase peptide fractionation kit. Fractions were dried using a SpeedVac concentrator and resuspended in 1% formic acid prior to LC-tandem MS (MS/MS) analysis.

### LC-MS/MS analysis.

The samples were analyzed on an Orbitrap Fusion Tribrid mass spectrometer with an Easy-nLC 1000 liquid chromatograph, a 75-μm by 2-cm Acclaim PepMap100 C_18_ trapping column, a 75-μm by 25-cm PepMap RSLC C_18_ analytical column, and an Easy-Spray ion source (Thermo Scientific). The column temperature was maintained at 45°C, and the peptides were eluted at a flow rate of 300 nl/min over a 110-min gradient, from 3 to 30% solvent B (100 min), 30 to 50% solvent B (3 min), 50 to 90% solvent B (2 min), and 90% solvent B (2 min). Solvent A was 0.1% formic acid in water, and solvent B was 0.1% formic acid in acetonitrile.

The full MS survey scan (*m/z* 400 to 1,500) was acquired in the Orbitrap at a resolution of 120,000 and an automatic gain control (AGC) target of 2 × 10^5^. The maximum injection time for MS scans was 50 ms. Monoisotopic precursor ions were selected with charge states 2 to 7, a ±10-ppm mass window, and 70-s dynamic exclusion. The MS^2^ scan (*m/z* 400 to 2,000) was performed using the linear ion trap with the collision-induced dissociation (CID) collision energy set to 35%. The ion trap scan rate was set to “rapid,” with an AGC target of 4 × 10^3^ and a maximum injection time of 150 ms. Ten fragment ions from each MS^2^ experiment were subsequently selected for an MS^3^ experiment. The MS^3^ scan (*m/z* 100 to 500) was performed to generate the TMT reporter ions in the linear ion trap using heated capillary dissociation (HCD) at a collision energy setting of 55%, a rapid scan rate and an AGC target of 5 × 10^3^, and a maximum injection time of 250 ms.

### Proteome data analysis.

All MS spectra were searched using the Proteome Discoverer (version 2.1.0.81; Thermo Scientific) with the Sequest-HT searching engines against an Aspergillus niger CBS 513.88 database containing 10,549 sequences (NCBI). The searches were performed with the following parameters: 5-ppm tolerance for precursor ion masses and 0.6-Da tolerance for fragment ion masses. The static modification settings included carbamidomethyl of cysteine residues and dynamic modifications included oxidation of methionine, TMTsixplex modification of lysine ε-amino groups and peptide N termini, and acetyl modification of protein N terminus. A target-decoy database search was used to set a false-discovery rate (FDR) of 1%. The reporter ion integration tolerance was 0.5 Da. The coisolation threshold was 75%. The average signal-to-noise threshold of all reporter peaks was bigger than 10. The total intensity of a reporter ion for a protein was calculated based on the sum of all detected reporter ions of associated peptides from that protein. The ratios between reporter ions and the reference reporter ions (TMT^6^-130 or -131) were used to estimate the abundance ratio of each protein.

For statistical analysis, the sum of reporter ion intensities for each protein was log_2_ transformed and the technical triplicate measurements for each protein were averaged. Only the proteins that were identified with at least one peptide detected in each technical replicate, and quantified in all three technical replicates, were considered for the analysis. Student’s *t* test was performed to identify proteins that are differentially expressed. Proteins with *P* values of <0.05 were further evaluated for increased or decreased abundance using a cutoff value of log_2_ fold change of >|1|.

### Data availability.

WGS data for JSC-093350089 are available in NCBI GenBank, under BioSample accession number SAMN06076678 and BioProject accession number PRJNA355122. Raw WGS reads are available in NCBI SRA, under accession number SRP127978. Proteomics data are accessible through the ProteomeXchange Consortium via PRIDE with the data set identifier PXD008588. The JSC-093350089 genome was annotated and uploaded in NCBI GenBank with accession number MSJD00000000.
